# The Qingchangligan Formula Alleviates Acute Liver Failure by Regulating Galactose Metabolism and Gut Microbiota

**DOI:** 10.3389/fcimb.2021.771483

**Published:** 2022-01-19

**Authors:** Ruiying Yin, Shuhui Liu, Xuejiao Jiang, Xiangying Zhang, Feili Wei, Jianhua Hu

**Affiliations:** ^1^ Beijing Youan Hospital, Capital Medical University, Beijing, China; ^2^ School of Traditional Chinese Medicine, Capital Medical University, Beijing, China; ^3^ Beijing Institute of Hepatology, Beijing Youan Hospital, Capital Medical University, Beijing, China

**Keywords:** gut microbiota, acute liver failure, traditional chinese medicine, Qingchangligan formula, THBS1

## Abstract

The Qingchangligan formula (QCLGF) is a traditional Chinese medicine that has significant clinical potential for patients with acute liver failure (ALF). However, the experimental evidence of the effect of QCLGF on ALF and the associated mechanisms remain elusive. We aimed to evaluate the function of QCLGF in ALF and the underlying mechanism. ALF was induced in rats by intraperitoneal injection of D-GalN (1100 mg/kg). The Qingchangligan formula was administered to the rats (6.725 g/kg · d) for 5 days, and the model group and the control group were given the same amount of physiological saline. Then 16S rRNA gene sequencing, high performance gas chromatography-mass spectrometry (GC-MS), and RNA-seq analysis were performed on the samples. The levels of ALT and AST in the ALF rats were abnormal (5322.08 ± 566.27 U/L and 7655.95 ± 1238.08 U/L, respectively) compared with the normal control (98.98 ± 6.90 U/L and 99.63 ± 10.94 U/L, respectively). The levels of ALT and AST in the QCLGF rats (2997.67 ± 469.24 U/L and 4158.40 ± 596.07 U/L, respectively) were closer the normal control group. Liver HE staining showed that the degree of liver damage in the QCLGF rats was lighter than that in the ALF rats. The overall structure of the gut microbiota after ALF was significantly altered, including *Proteobacteria*, *Blautia*, *Romboutsia*, *Parabacteroides*, *UCG-008*, *Parasutterella*, *Ruminococcus*, *norank_f:Lachnospiraceae*, the *Eubacterium_xylanophilum_group*, *Oscillibacter*, and *Eisenbergiella*. QCLGF balanced the structure and abundance of intestinal flora. The levels of D(+)galactose, isopropyl beta-D-1-thiogalactopyranoside and D-mannitol were lighter in the plasma of the ALF rats than in the normal control rats, but there were significantly elevated levels of those metabolites in the QCLGF rats. The gene expression changed significantly in the ALF rats. QCLGF regulated the expression of THBS1 and the KEGG pathways of carbohydrate metabolism, lipid metabolism, signal transduction, the immune system, and infectious disease: bacterial. QCLGF may alleviating intestinal flora disorder, regulating galactose metabolism and downregulating the expression of THBS1 to alleviate D-GalN induced acute liver failure.

## Introduction

Acute liver failure (ALF) is a severe consequence of abrupt hepatocyte injury that can evolve over days or weeks to a lethal outcome. The five most prevalent causes of ALF are acetaminophen overdose, viral hepatitis, drug-induced liver injury, Wilson’s disease, and autoimmune hepatitis (Rajaram and Subramanian, 2018). The management of patients with ALF includes general considerations, cause-specific management, administration of drugs to alleviate liver failure, management of systemic complications of acute liver failure, and liver transplantation (Stravitz and Lee, 2019). Few therapeutic options are available for ALF, but study has shown that protection from liver injury is possible with early administration of acetylcysteine when paracetamol overdose is suspected (Fisher and Curry, 2019). Acetylcysteine mainly replenishes glutathione reserves depleted in APAP detoxification. However, this intervention is only partially effective and is accompanied by adverse effects, including anaphylactoid reaction (Yarema et al., 2018). Therefore, it is necessary to find other interventions for acute liver failure patients.

Evidence suggests that the degree of liver damage is reduced when intestinal bacteria that may be connected with gut bacteria derived liver inflammation are removed (Gong et al., 2018; Castillo-Dela et al., 2019). Microbiome-mediated upstream signals may regulate the gene expression of MYC during ALF, resulting in liver injury (Kolodziejczyk et al., 2020). However, some probiotics can reduce the extent of liver damage, such as *Lactobacillus salivarius* LI01 and *Pediococcus pentosaceus* LI05, that significantly reduce the elevated alanine aminotransferase and aspartate aminotransferase levels in ALF rats, prevent an increase in total bilirubin, decrease the histological abnormalities of the liver, and change the structure of the cecal microbiome (Lv et al., 2014; Shi et al., 2017; Zhuge et al., 2020). Metabolites of intestinal flora such as 1-phenyl-1,2-propanedione intensify liver injury by depleting hepatic glutathione, an important antioxidant (Gong et al., 2018). Metabolites of gut microbiota, including hexanoic acid, trigeminal, 1-hexadecanol, campesterol, d-lactose, and lithocholic acid, are significantly changed in ALF rats, and those may affect the degree of liver injury (Jiang et al., 2021).

QCLGF is a traditional Chinese medicine (TCM) comprised of five herbs: Rheum palmatum, dried Rehmannia root, Magnolia Officinalis, Taraxacum Officinale, and Fructus Aurantii Immaturus. QCLGF has been applied in clinical practice for decades and we have previously demonstrated that QCLGF is practical and effective in the prevention and treatment of liver failure (Hu and Li, 2017). Previous studies have demonstrated that QCLGF might be involved in repressing inflammatory factors, improving systemic and hepatic recovery by modulating autophagy and the MAPK signal pathway (Zhang et al., 2017; Ding et al., 2018).

TCM often contains compounds of different structural types. Chinese herbal compounds are often multi-component and multi-target. So, the effect of QCLGF may not be limited to the pathways we previously discovered. Most TCM contains fiber, polyphenols and polysaccharides, exerting prebiotic-like activity to maintain the health of intestinal microecology (Khan et al., 2018; Sun et al., 2019). Previous studies have suggested that the production and biological activity, and oral bioavailability of plant polyphenols widely contained in TCM are often less than 10%, which is modulated by gut microbiota (Cardona et al., 2013; Kawabata et al., 2019). We consider it necessary to study further about how QCLGF affects the structure and richness of intestinal flora in liver injury.

We chose D-GalN as the hepatotoxic drug to induce ALF (Lv et al., 2014). By investigating the efficacy of QCLGF treatments, the structure of the gut microbiota, the metabolites of plasma, and the gene expression of the liver, we identified that QCLGF potentially alleviated ALF by modulating gut microbiota and affecting lactose metabolism. Our finding provides crucial experimental evidence that QCLGF provides protection from ALF, and reveals other pathways of the curative effect of QCLGF.

## Results

### QCLGF Protects Rats From D-GalN-Induced Liver Injury

**Figure 1:**
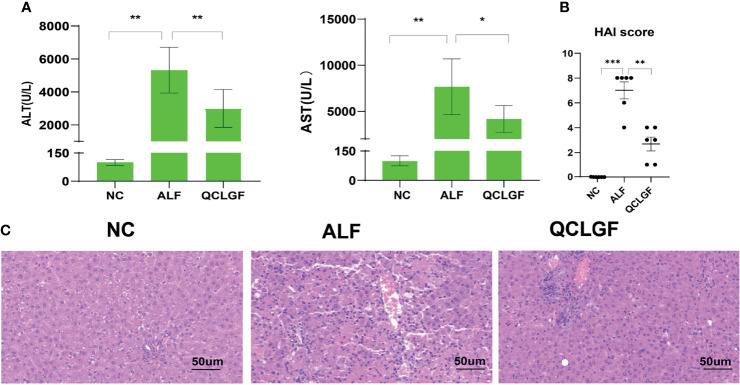
QCLGF alleviates d-galactosamine-induced liver injury. **(A)** Plasma ALT and AST levels were determined 12 h after D-GalN intraperitoneal injection. **(B)** Liver HAI score. **(C)** Representative liver sections from different treated groups (*n* = 6, per group). (*P < 0.05, **P < 0.01 and ***P < 0.001 compared with the ALF group, Wilcoxon rank sum test).

First, we evaluated whether QCLGF could attenuate liver injury induced by D-GalN in rats. The plasma ALT and AST levels were significantly higher at 24 hours after injecting D-GalN compared with the normal SD rats (NC group). And the plasma ALT [NC vs ALF, *P* < 0.01, ALF vs QCLGF, *P* < 0.01, Wilcoxon rank sum test] and AST [NC vs ALF, *P* < 0.01, ALF vs QCLGF, *P <* 0.001, Wilcoxon rank sum test] levels were significantly higher in ALF group compared with the SD rat treated with QCLGF group (**Figure 1A**).

Histopathological analysis reaffirmed these results (**Figures 1B, C**), demonstrating that QCLGF significantly reduced liver damage in rats. There were no abnormal differences in histological changes in the liver of normal rats. The hepatic lobules were clear and no degeneration or necrosis of hepatocytes was observed. In contrast, D-GalN treatment caused severe damage in the livers of the rats, as demonstrated by massive hepatocyte necrosis, inflammatory cell infiltration, and hemorrhage. Pretreatment with QCLGF markedly attenuated liver damage in the rats and reduced these typical histological changes.

### Community Structure of Gut Microbiota Shows Distinct Changes After Different Treatments

**Figure 2:**
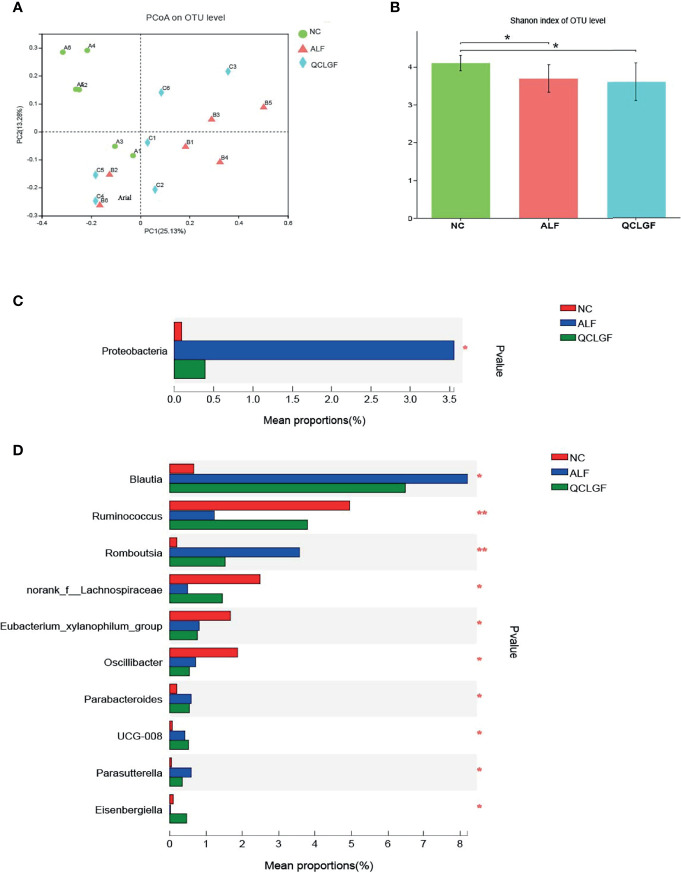
QCLGF protects against D-galactosamine-induced beneficial bacterium depletion and pathogen enrichment. **(A)** Beta diversity was determined by the Bray–Curtis distance-based principal. **(B)** Shannon index describing the alpha diversity of the microbiota in the three groups, ALF and normal groups (Shannon, *P*_adj_ = 0.04, t-test), QCLGF and ALF (Shannon, *P*_adj_ = 0.049, t-test). **(C)** Alterations in the relative abundances of bacterial taxa at phylum levels in QCLGF, NC, and ALF groups. (*P < 0.05, one-way ANOVA). **(D)** Alterations in the relative abundances of bacterial taxa at genus levels in QCLGF, NC, and ALF groups. (*P < 0.05 and **P < 0.01, one-way ANOVA).

The structure of the intestinal flora in the different treatment groups was different. Specifically, in the Bray–Curtis distance-based principal coordinate analysis (PCoA), the gut microbiota structure of the ALF groups showed a distinct deviation along PCoA1 (explaining 25.13% of the variation) with QCLGF, indicating significant changes in the core microbiota after the treatments (**Figure 2A**).

The structure of the gut microbiota showed significant shifts in the ALF and normal groups (Shannon, *P*_adj_ < 0.05, t-test). The structure of the gut microbiota showed substantial changes in the QCLGF and NC group (Shannon, *P*_adj_ < 0.05, t-test) groups. The microbial structure of the ALF group resembled that of the QCLGF treatment group (Shannon, *P*_adj_ = 0.732, t-test) (**Figure 2B**).

We next explored the variations before and after treatment at the hylum and genus levels. Proteobacteria was increased at the phylum level in the ALF group compared to the NC group and the QCLGF group (*P* < 0.05, one-way ANOVA) (**Figure 2C**). According to clustering analysis of all genera based on changes in abundance, we showed that D-GalN treatment markedly altered the structure of the gut microbiota, and enriched several genera, including *Blautia*, *Romboutsia*, *Parabacteroides*, *UCG-008*, and *Parasutterella*. But, *Ruminococcus*, *norank_f_Lachnospiraceae*, the *Eubacterium_xylanophilum_group*, *Oscillibacter*, and *Eisenbergiella* were reduced. *Blautia*, *Romboutsia*, *Parabacteroides*, *Parasutterella*, *Ruminococcus*, *norank_f_Lachnospiraceae*, and *Eisenbergiella* on genus, were close to normal in QCLGF group. (*P < 0.05, **P < 0.01, one-way ANOVA) (**Figure 2D**).

### QCLGF Relieves D-Galactosamine-Induced Plasma Metabolic Disorders

**Figure 3:**
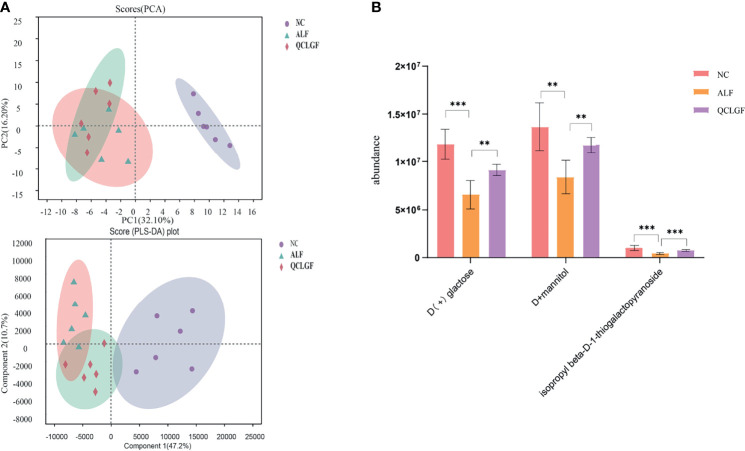
QCLGF protects against D-galactosamine-induced altered plasma metabolites. **(A)** Changes in the ion intensity of three markers with beta-D-1-thiogalactopyranoside, D(+)galactose, and D-mannitol (t-test, **P_adj_< 0.01,***P_adj_< 0.05). **(B)** Principal component analysis (PCA) (PC1 = 32.1%). And the partial least squared discriminant analysis (PLS-DA) score plot based on GC-MS profiling data of plasma samples (compound1 = 47.2%). Each dot with three kinds of color represents the different samples.

We next measured the plasma metabolite levels. A total of 193 metabolites were identified by gas chromatography-mass spectrometry (GC-MS), which could discriminate between different groups according to their different peak areas of metabolites, to reveal the intervention effects of QCLGF. As shown in **Figure 3A**, score plots of principal components analysis PC1 (32.10%) and partial least squares-discriminant analysis Component 1 (47.2) showed that the metabolome profiles of the N group, the ALF group, and the QCLGF group were separately clustered, and the plasma metabolic profiles among the three groups were significantly different. The metabolites of the ALF group and the normal control group changed significantly.

Student’s t-test was used to statistically analyze the metabolites variation tendencies of acute liver failure. We found that 20 metabolites changed significantly between the normal control group and the ALF group (VIP > 1, FDR < 0.05) (**Table 1**). Then we found that the levels of isopropyl beta-D-1-thiogalactopyranoside, D(+)galactose, and D-mannitol were close to those of the normal control groups (*P_adj_ < 0.05, **P_adj_ < 0.01, ***P_adj_ < 0.001, Student’s t-test) after treatment with QCLGF. Those metabolites play a key role in the treatment of liver failure by QCLGF (**Figure 3B**).

**Table 1:** We found that 20 metabolites changed significantly between the normal control group and the ALF group (VIP > 1, FDR < 0.05, *n* = 6).

Metabolite	VIP	FC(ALF/NC)	P_value	FDR
naphthalene	4.78	0.02	0.01	0.02
D-mannitol	4.66	0.35	0.00	0.00
D(+)galactose	4.52	0.32	0.00	0.00
phosphoric acid	4.33	0.66	0.00	0.00
ethanolamine	4.05	0.59	0.00	0.00
Urea	2.57	0.72	0.02	0.03
digalacturonic acid	2.19	0.25	0.00	0.00
sarcosine	2.03	0.60	0.00	0.00
tyrosine	1.78	3.93	0.00	0.01
L-proline	1.70	0.59	0.00	0.01
L-lysine	1.41	0.28	0.00	0.00
oxalic acid	1.40	0.62	0.00	0.00
palmitic acid	1.37	2.09	0.00	0.00
Isopropyl beta-D-1-thiogalactopyranoside	1.36	0.26	0.00	0.00
2-piperidone	1.23	0.60	0.00	0.00
L-threonine	1.13	0.63	0.00	0.01
1-decanol(decyl alcohol)	1.06	0.73	0.01	0.02
5-aminovaleric acid	1.05	0.73	0.02	0.04
Methamphetamine	1.03	0.58	0.00	0.00
2-hydroxypyridine	1.00	0.62	0.00	0.00

### Gene Expression Profiles of Rat Liver

**Figure 4:**
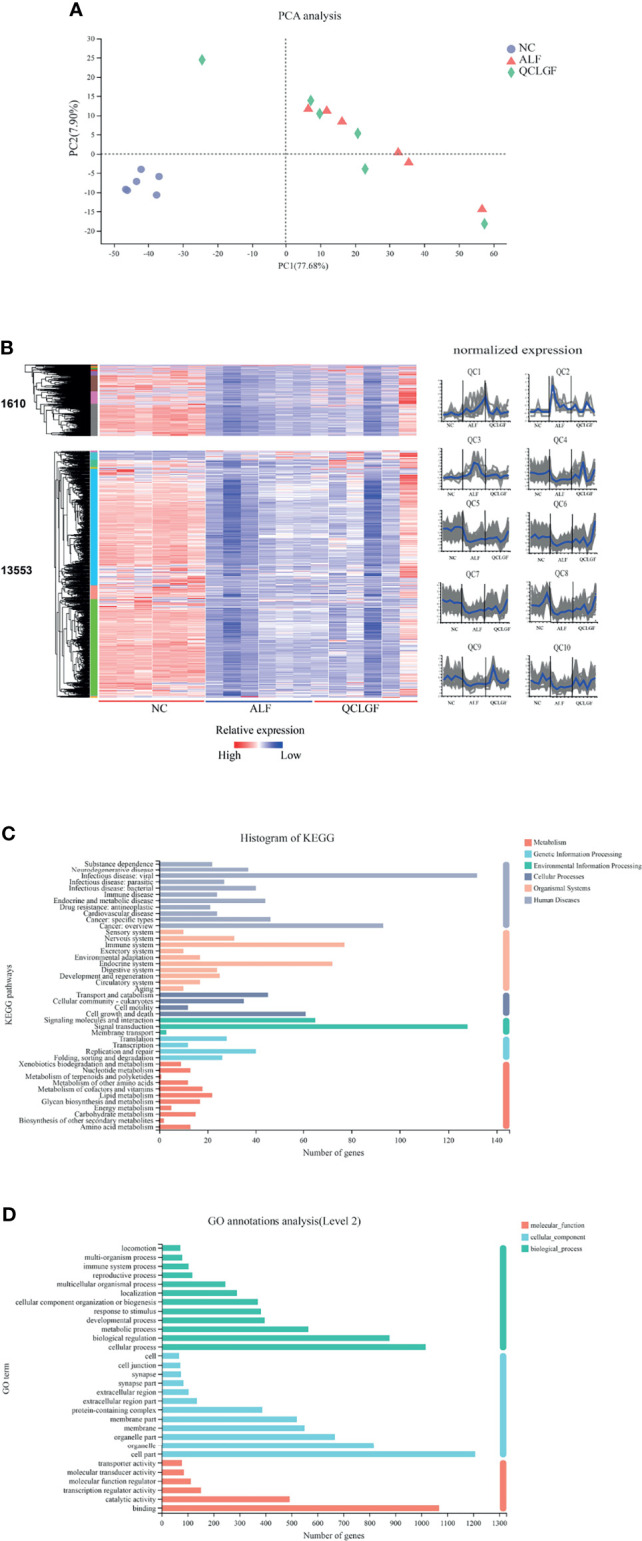
Comparisons of gene expression profiles in the rat liver by RNA-seq among different treatment groups. **(A)** Principal component analysis showed that the NC group had distinct gene expression signatures compared with the ALF rats and QCLGF rats PC1 (explaining 77.68% of variation). **(B)** Heatmap of DEGs. Compared with the NC group, the number of differentially expressed genes (DEGs) in the ALF groups was 13553 (DEG1, 529/13024; upregulated and downregulated DEGs, DEGseq, respectively). Genes of QCLGF vs. ALF and NC vs. ALF with the same trend were 1610 (DEG3, 1581/29; upregulated and downregulated DEGs, DEGseq, respectively), DEG3 genes were further clustered into 10 QCLGF clusters (QCs) according to their expression profiles. **(C)** KEGG annotation of DEG3. **(D)** GO annotation analysis of DEG3.

Next, we used transcriptomic analysis to determine whether the gene expression profiles of the rat livers were similar between different treatment groups. Principal component analysis showed that NC group had distinct gene expression signatures compared with ALF rats or QCLGF rats PC1 (explaining 77.68% of variation). Then, we set out to identify genes that were differentially expressed through pairwise comparisons of groups (FC > 1.2, *P*_adj_ < 0.05). Compared with NC group, the number of differentially expressed genes (DEGs) in the ALF group was 13553 (DEG1, 529/13024; upregulated and downregulated DEGs, DEGseq, respectively). Compared with the ALF group, the number of differentially expressed genes (DEGs) in the QCLGF group was 6455 (DEG2, 6351/104; upregulated and downregulated DEGs, DEGseq, respectively). Genes of QCLGF vs. ALF and NC vs. ALF with the same trend were 1610 (DEG3, 1581/29; upregulated and downregulated DEGs, DEGseq, respectively), DEG1 and DEG3 of genes were further clustered into nine ALF clusters (ACs) and nine QCLGF clusters (QCs) according to their expression profiles (**Figures 4A, B**). QC1, QC2 and QC3 displayed activated expression patterns in the ALF group compared to the NC group and the QCLGF group, while QC4, QC5, QC6, QC7, QC8, QC9 and QC10 were repressed in the ALF group compared to the NC group and the QCLGF group.

DGE3 was subjected to both KEGG annotation and Gene Ontology (GO) term annotation analyses (**Figures 4C, D**). KEGG annotation of the significantly differentially expressed genes showed that more genes were annotated into the carbohydrate metabolism, lipid metabolism, signal transduction, immune system, and infectious disease: bacterial. GO annotation was mainly involved in the metabolic process, biological regulation and cellular process.

### Quantitative Real-Time RT-PCR

**Figure 5:**
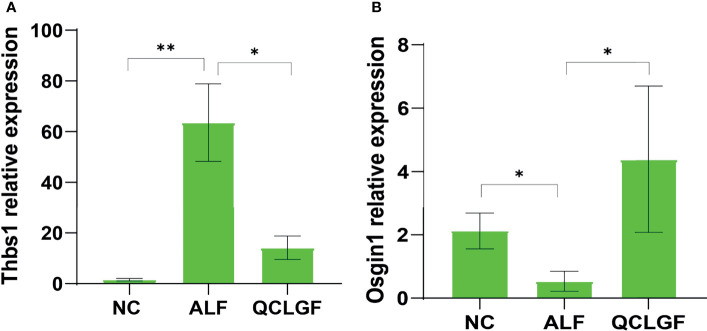
Analysis of expression of DEG genes in rat liver using RT-qPCR. Expression levels of two candidate genes, Osgin1 **(A)** and Thbs1 **(B)** were measured. Wilcoxon ranksum test was applied to test the differences in gene expression levels among the three groups. (All data was presented as Mean ± SEM, *P < 0.05; **P < 0.01. *n* = 6 samples per group).

To verify the differences in genes, real-time fluorescence quantitative PCR was conducted. The expression levels of THBS1 were significantly elevated in the ALF group compared with the NC group (*P* < 0.01, Wilcoxon rank sum test). The expression of THBS1 was significantly downregulated in all samples pre-treated with QCLGF (*P* < 0.05, Wilcoxon rank sum test). The expression levels of OSGIN1 were significantly downregulated in the ALF group (*P* < 0.05, Wilcoxon rank sum test). The expression levels of OSGIN1 were significantly upregulated in all samples pre-treated with QCLGF (*P* < 0.05, Wilcoxon rank-sum test) (**Figure 5**).

## Discussion

Our study was consistent with a previous study showing that QCLGF could prevent the development of liver injury. Our study showed there is a significant difference in gut microbiota in normal control rats compared to ALF rats, suggesting the diversity of intestinal flora decreased in acute liver failure. It is of note that the gut microbiota of the QCLGF pretreatment group was not significantly different from that of the ALF rats, showing that QCLGF only adjusted part of the gut microbiota but could not completely reverse the disorders in the gut microbiota of the ALF rats. The relative abundance of the *Proteobacteria* phylum increases in acute liver failure, and QCLGF regulates this disorder. Similarly, both individuals with liver cirrhosis and those with non-alcoholic fatty liver disease have an enrichment of the *Proteobacteria* phylum (Qin et al., 2014; Shen et al., 2017). Another study has shown that the abundance of *Proteobacteria* is a sign of intestinal flora disorder, inducing metabolic disturbances of the host. So, that means QCLGF may reduce acute liver failure by regulating the abundance of opportunistic pathogens.

Blautia, Romboutsia, Parabacteroides, UCG-008 and Parasutterella genera were enriched and Ruminococcus, norank_f:Lachnospiraceae, Eubacterium_xylanophilum_group, Oscillibacter and Eisenbergiella genera were reduced in the ALF group. The significantly changed genus may be the hallmark bacteria of acute liver failure. Similarly, Blautia increased in stool in patients with nonalcoholic steatohepatitis (Shen et al., 2017). Parasutterella expression was related to chronic intestinal inflammation (Chen et al., 2018). Oscillibacter is reduced in obese people (Thingholm et al., 2019). QCLGF improved disturbed intestinal flora in acute liver failure rats, especially increased the abundance of Ruminoccocus significantly. which bromides hydrolysis non-digestible dietary polysaccharide and short chain fatty acid causes the release of nutrients such as glucose that may reach bacterial species within the mucus layer, potentially impacting other species growth and maintaining the homeostasis of intestinal flora, that plays an important role in decreasing fat accumulation in the liver and maintaining intestinal barrier function to reduce LPS levels in circulating blood. (Latham and Wolin, 1977; Crost et al., 2018; Iwao et al., 2020; Takeuchi et al., 2021). On the one hand, we think that QCLGF regulates the structure and abundance of intestinal microbiota and balances energy metabolism in the gut by maintaining homeostasis between intestinal microbiota communities. On the other hand, QCLGF reduces gram-negative bacteria such as proteobacteria to reduce the chance of LPS entering the circulating blood.

We found significant changes in amino acids, fatty acids and saccharides in ALF rats. Such as, the increased of Tyrosine and palmitic acid increased, and the decreased of l-proline, L-Lysine and L-threonine. These indicate abnormal carbohydrate metabolism in ALF. Compared to the normal group, the ALF group showed significantly less beta-D-1-thiogalactopyranoside, D(+)galactose, and D-mannitol in plasm, indicating abnormal glucose metabolism in rats with acute liver failure. In acute liver failure, hepatocytes die in large numbers, but the patient is in a hypermetabolic state (Walsh et al., 2000). Isopropyl beta-D-1-thiogalactopyranoside is a lactose mimic that induces the transcription process of the lactose operon in prokaryotes. Mannitol may decrease the toxic effect of acetaminophen on the liver (Kyle et al., 1987; Celikmen et al., 2016). Galactose can be directly absorbed by liver cells, and the glucose 1-phosphate can be generated into the glucose metabolism pathway for energy supply by the action of galactokinase-galactose-1-phosphouridyl transferase and uridine diphosphate galactose-isomerase, successively. QCLGF can increase the level of isopropyl beta-D-1-thiogalactopyranoside, D(+)galactose, and D-mannitol in plasma. This suggests that QCLGF reduces the degree of liver injury by carbohydrate metabolism.

To determine the effects of QCLGF, we not only performed plasma metabolomics but also made an analysis of rat liver gene expression from different treatment groups. Compared to normal rats, ALF rats had different gene expression profiles. Treatment with QCLGF significantly altered liver gene expression by upregulating and downregulating specific sets of genes. Among the GO terms and KEGG pathways that were significantly enriched by QCLGF pretreatment were those involved in carbohydrate metabolism and apoptosis, which were downregulated. In addition, compared to normal rats, ALF rats had distinct plasma metabolomic profiles. The metabolomics of rats pretreated with QCLGF were more similar to normal rats.

Oxidative stress-induced growth inhibitor 1 (OSGIN1) and Thrombospondin 1(THBS1) were among three groups identified by qRT-PCR. NRF2-regulated OSGIN1 to make P53 accumulate and translocate to the nucleus (Brennan et al., 2017). P53 is a protein target downstream of NRF2 and cooperates to protect cells against oxidative damage (Faraonio et al., 2006). Thus, QCLGF may protect liver cells against oxidative stress by upregulated OSGIN1. Furthermore, THBS1 was found to play an important role in glucolipid metabolism. The downstream targets of THBS1 include ECM proteins and cell surface receptors. One receptor is transforming growth factor-β1 (TGF-β1), which plays a critical role in fibration and inflammation, exacerbating hepatic steatosis, fibrosis and failure (Breitkopf et al., 2005; Bai et al., 2020; Jefferson et al., 2020). The upregulation of THBS1 can be stimulated by glucose, and the overexpression of THBS1 contributes to obesity-induced tissue inflammation and the development of metabolic syndrome (Wang et al., 2003; Wang et al., 2004; Matsuo et al., 2015).

Collectively, these data suggest that QCLGF alleviates liver injury effects mainly by adjusting gut microbiota and the galactose metabolism. However, our study has several limitations. Firstly, we merely proved the role of QCLGF in alleviating liver injury, but we did not validate components of QCLGF. In the future, we should produce a complete spectrum of the components of QCLGF to standardize QCLGF, thus avoiding the unintended side effects of some components and identifying potentially active ingredients. Secondly, we haven’t added sterile rats as a control group, so whether or not the efficacy QCLGF is affected by gut microbiota is not clear. Next we may join experiments that use sterile animal models as vectors to verify the effect of intestinal bacteria on QCLGF.

## Materials and methods

### Reagents

QCLGF was obtained from Beijing Tongrentang Drugstore. The QCLGF was comprised of 5 Chinese medicinal materials, including Rheum palmatum, dried rehmannia root, Magnolia officinalis Rehd et Wils, Taraxacum officinalae and Fructus aurantii, and the dosage of each medicine was 15g. D-Galactosamine was obtained from Sigma-Aldrich (St. Louis, USA; cat: G0500).

### Animals

Male and female SD rats, all 8 weeks of age, were maintained under standard specific pathogen-free conditions and fed a normal rodent diet for 4 weeks.

### Experimental Design

After four weeks of acclimatization, 18 SD rats (male and female, 12 weeks old) were randomly divided into four groups: (1) NC group (*n* = 6), pregavage of normal saline (1 ml/100g); (2) ALF group (*n* = 6), pregavage of normal saline (1ml/100 g); (3) QCLGF group (*n* = 6), pregavage of QCLGF (1 ml/100 g). The duration of interventions lasted for 5 days. Three rats were kept in each cage. Oral gavage once per day. The N, ALF, and QCLGF groups acted as the normal control group, disease model group, and QCLGF group, respectively. After 5 days, intraperitoneal injection of D-GalN (1.1 g/kg, dissolved in normal saline, with the pH adjusted to 7.2 with sodium hydroxide) was administered to the ALF group and the QCLGF group. After 24 hours, all the animals were anesthetized with diethyl ether, and specimens were quickly collected including abdominal aorta blood plasma, the left hepatic lobe, and cecal contents.

### Assessment of Liver Damage

Plasma alanine aminotransferase (ALT) and aspartate aminotransferase (AST) were determined using a multi-parametric analyzer (Chemray 240, Chemray 800, rayto, China). 1×1 cm liver tissues were cut from the left lobe, fixed in 4% formaldehyde, embedded in paraffin, sectioned, and stained with H&E. Pathological hepatic tissue damage was evaluated by HAI scoring (Knodell R. G. et al., 1981).

### 16S rRNA Gene Sequence Analysis

Each cecal contents sample was snap frozen in liquid nitrogen within minutes of donation and then kept at −80°C. Genomic DNA was extracted by a Feces DNA Kit from the cecal contents. The V3-V4 hypervariable regions of the bacteria 16S rRNA gene were amplified by PCR instrument (ABI GeneAmp^®^ 9700, USA) with barcode-indexed primers 338F (5’-ACTCCTACGGGAG GCAGCAG-3’) and 806R (5’-GGACTACHVGGGTWTC TAAT-3’). The purified amplicons were pooled in equimolar concentration and paired-end sequenced on an Illumina Miseq platform (Illumina, San Diego, California, USA). Purified amplicons were pooled in equimolar and paired-end sequenced (2 × 300) on an Illumina MiSeq platform according to the standard protocols by Majorbio Bio-Pharm Technology Co. Ltd. (Shanghai, China).

### Determination of Plasma Metabolites

Plasma metabolite levels were measured by GC-MS on the Agilent 8890B-5977B Gas-Chromatography-Mass Spectrometry Instrument (Agilent, USA). Sample by DB-5MS capillary column (40m × 0.25mm × 0.25µm, Agilent 122-5532G) separated into mass spectrometry. The chromatographic conditions: The injection port temperature was 260°C, the carrier gas was high purity helium, the carrier gas flow rate was 1 mL/min, the spacer purge flow rate was 3 mL/min, and the solvent was delayed 5.5 min. The operation conditions of the mass spectrometer were as follows: Electron bombardment ion source (EI), transmission line temperature was 310°C, ion source temperature was 230°C, quadrupole temperature was 150°C, electron energy was 70 eV. The SCAN mode was full SCAN mode. The SCAN range was from 50 to 500 m/Z. The SCAN frequency was 3.2 scan/s. MassHunter was used for the offline file Workstation Quantitative Analysis (V10.0.707.0) to obtain metabolite identification results and data matrix, combined with t-test and VIP (OPLS-DA) to screen the differential metabolites.

### RNA-Seq Analysis of the Liver

Total RNA from liver tissue samples was extracted with TRIzol (Catalog No. 15596026; ThermoFisher, USA) according to the standard isolation protocol. The RNA libraries for sequencing were constructed using the VAHTS Universal V6 RNA-seq Library Prep Kit for Illumina (Catalog No. NR604-01) with 2 ug total RNA according to the manufacturer’s protocol. In brief, poly(A) + mRNA was enriched from the total RNA using the mRNA Capture Beads, and then the purified mRNA was randomly fragmented into sequences approximately 400 bp in length. A cDNA library was obtained with random hexamer primers. After the end repair of the cDNA fragments, adaptors were added to the other end of the cDNA products, and then the cDNA library was amplified by PCR. After validation by qPCR, libraries were finally sequenced on the Illumina HiSeqXTen platform using the PE150 module. DEGs were identified using the DESeq2 program with the cutoff threshold of *P* < 0.05 and the absolute value of log2 fold change (log2 FC) > 1. GO (http://www.geneontology.org/) and KEGG enrichment analyses (http://www.genome.jp/kegg) were performed using DEGs as the foreground genes and all genes as the background.

### Real-Time Quantitative PCR

Total RNA was isolated from the liver using TRIzol reagent (Catalog No. 15596018; ThermoFisher) and then transcribed to cDNA using a Strand cDNA Synthesis Kit (Catalog No. 6210A; Takara). RT-qPCR was performed with LightCycler 480, and technical triplicates using TB Green reagent (Catalog No. RR420A; Takara). The expression levels were calculated with the 2^^(-ΔΔCT)^ method, and the CT values were normalized using Gapdh as a reference gene. The target genes were Thbs1 and Osgin1.

### Statistical Analysis

Statistical analyses were performed using GraphPad 7.0. Data are presented as the Mean ± SEM. The t test or Wilcoxon rank-sum test was used for comparisons between groups when appropriate. Bray–Curtis distance was calculated as the beta diversity measurement using the vegan package. The PERMANOVA test with the adonis package was used to calculate the differences in the structure of the community. The Capscale package was used to perform the PCoA of all samples based on the Bray–Curtis distance. *P* < 0.05 or BH-adjusted *P* < 0.05 was considered statistically significant. Metabolites were tentatively assigned by molecular formula matching and related information obtained from online databases such as the Human Metabolome Database (HMDB, http://www.hmdb.ca/spectra/ms/search) (Wishart et al., 2018). Pathway analysis was performed on the KEGG website (Kanehisa et al., 2017) (http://www.genome.jp/kegg/).

## Data Availability Statement

The datasets presented in this study can be found in online repositories. The names of the repository/repositories and accession number(s) can be found below: NCBI SRA BioProject, accession numbers: PRJNA790471, PRJNA790477.

## Ethics Statement

The animal study was reviewed and approved by Capital Medical University. Ethical code: AEEI-2020-160.

## Author Contributions

RY, JH, and FW conceived and designed the experiments. JH, SL, XZ, and XJ were involved in the experimental study design, preparation, and review of this manuscript. All authors contributed to the article and approved the submitted version.

## Funding

This research was funded by the Key medical major of Beijing sailing plan, severe liver disease with integrated traditional Chinese and Western medicine (No. zylx201819).

## Conflict of Interest

The authors declare that the research was conducted in the absence of any commercial or financial relationships that could be construed as a potential conflict of interest.

## Publisher’s Note

All claims expressed in this article are solely those of the authors and do not necessarily represent those of their affiliated organizations, or those of the publisher, the editors and the reviewers. Any product that may be evaluated in this article, or claim that may be made by its manufacturer, is not guaranteed or endorsed by the publisher.
